# COVID-19 Limitations on Doodling as a Measure of Burnout

**DOI:** 10.3390/ejihpe11040118

**Published:** 2021-12-16

**Authors:** Carol Nash

**Affiliations:** History of Medicine Program, Department of Psychiatry, Temerty Faculty of Medicine, University of Toronto, Toronto, ON M5S 1A1, Canada; carol.nash@utoronto.ca

**Keywords:** COVID-19, burnout, doodling, team mindfulness, anxiety, depression

## Abstract

Pre-COVID-19, doodling was identified as a measure of burnout in researchers attending a weekly, in-person health narratives research group manifesting team mindfulness. Under the group’s supportive conditions, variations in doodling served to measure change in participants reported depression and anxiety—internal states directly associated with burnout, adversely affecting healthcare researchers, their employment, and their research. COVID-19 demanded social distancing during the group’s 2020/21 academic meetings. Conducted online, the group’s participants who chose to doodle did so alone during the pandemic. Whether the sequestering of group participants during COVID-19 altered the ability of doodling to act as a measure of depression and anxiety was investigated. Participants considered that doodling during the group’s online meetings increased their enjoyment and attention level—some expressed that it helped them to relax. However, unlike face-to-face meetings during previous non-COVID-19 years, solitary doodling during online meetings was unable to reflect researchers’ depression or anxiety. The COVID-19 limitations that necessitated doodling alone maintained the benefits group members saw in doodling but hampered the ability of doodling to act as a measure of burnout, in contrast to previous in-person doodling. This result is seen to correspond to one aspect of the group’s change in team mindfulness resulting from COVID-19 constraints.

## 1. Introduction

Burnout—a negative, job-related psychological state exhibited through physical fatigue, emotional exhaustion, and loss of motivation [1]—is a syndrome arising from prolonged chronic interpersonal stressors associated with work. It is represented by three key dimensions [2]: overwhelming exhaustion, negative work-related feelings of cynicism and disassociation, and a sense of futility from perceived job-affiliated failure. Furthermore, it has been particularly associated with the health professions [3]. Early in the history of burnout research, it was found to be significantly related to job termination but not to absenteeism [4]. For healthcare researchers and their employers, the discontinuation of work undertaken equates to a loss of the production of valuable research [5]. Directly associated with symptoms of depression and anxiety [6,7], if burnout is to be diminished, an easily employed and reliable measure of depression and anxiety is important.

In contrast to burnout, work engagement is a positive and fulfilling state of mind related to work characterized by behaviors that are vigorous, dedicated, and absorbed in the task at hand [8]. Work engagement has a positive influence on work-related performance [9]. Embedded in their work environment, positive individual attitudes and behavioral expressions of researchers in a work organization are interacting results of these individuals’ own attributes and work-defined factors, which include the interpersonal relationships among team members [10]. Team mindfulness, with respect to the construction and maintenance of work environments, has become an increasingly relevant consideration regarding enhanced, work-related, personal fulfilment [11]—where team mindfulness is defined as a common belief among team members that their group’s cohesiveness is based on non-judgmental awareness and attention in realizing team-related experiences [12].

Doodling is defined as aimless scrawl made while a person’s attention is otherwise engaged [13]. Reasons for this behavior have been identified as boredom, the need for a productive activity while otherwise engaged, a form of fidgeting when forced to stay inactive, a means of artistic expression [14], to provide “thinking” benefits [15], as a method of discovery [16], or as something, when compared with coloring and free drawing, that is paramount in activating the medial prefrontal cortex [17]. What doodling behavior is not is a method to improve task-related episodic memory [18].

Doodling has recently been recognized as a potential measure of depression and anxiety based on unexpected outcomes from comparing doodles over a number of years, associated with one discretional health narrative research group supporting diversity of membership where doodling was introduced [19]. The result regarding casual, self-reported levels of depression and anxiety of group participants indicated—under the well-defined conditions of the group—that modifications in doodling over the sessions offered a measure of change in these internal states of the researchers. As such, there is reason to suppose that doodling holds the potential to directly appraise the range of disaffect associated with burnout and increase work engagement in researchers under conditions where team mindfulness is supported and maintained.

In considering whether doodling is able to act as a reliable measure of internal states during COVID-19, it should be noted that there has been little academic research in general concerning doodling. The first peer-reviewed research on doodling was a study of over 9000 doodlers before WWII [20], the next controlled study of doodling was not undertaken until seventy years later when doodling by nurses was considered [21]. Subsequently, a test was created and tested for doodling [22], and the *Lancet* published a study regarding doodling [23]. Since then, the number of studies of doodling has increased [24,25,26,27,28,29,30] and, in 2015, the 2009 test was replicated [31]. Nevertheless, in over eighty years, these remain doodling’s peer-reviewed studies—none of which had considered doodling as a valid measure of the psyche until the report on the Health Narratives Research Group from last year [19].

The positive results of doodling’s ability to measure the affective level of healthcare researchers in relation to one health narratives research group were demonstrated when the group met in person during the pre-COVID-19 years. The group operated as a mindful team [12], respectful of each member and encouraging of each person’s point of view. This was done by the group as a whole, regularly attending to the team members’ experiences in a non-judgmental way [32]. The group facilitator recorded the full transcript of the group’s activities and posted it to a private Facebook group (to which every participant was a member), ensuring that the views of each participant were given the attention envisioned when they were revealed to the group. The group then continued entirely online for a full year after the pandemic social distancing limitations, during the 2020/21 academic year. Other than the group migrating to an online meeting format, all other aspects of this health narratives research group remained as they were during previous years. Yet, the lack of in-person meetings altered the team mindfulness and resulted in a very noticeable difference with respect to doodling. With the change in this one important variable, it was useful to compare the ability of doodling to gauge internal states when the doodling was done alone during the pandemic year, rather than in person with others as in years before.

If doodling retained its ability to identify the researchers’ depression and/or anxiety status when meetings were online, then there is reason to believe it represents a robust measure. Otherwise, the usefulness of doodling for reflecting internal states may be dependent on in-person meetings under the specific conditions of this health narratives research group where team mindfulness was supported. To determine the outcome, feedback from the participants in the group during the spring session of the 2020/21 academic year will be presented, and the results of the previous in-person meetings of the group will be compared with the doodles produced alone during the COVID-19 pandemic for those researchers who acknowledged experiencing continuing research-related depression and anxiety.

## 2. Materials and Methods

The Health Narratives Research Group (HeNReG), offered through the Department of Psychiatry at the University of Toronto, is designed to take each participant’s story that initiated their healthcare interest and, with the help of weekly writing prompts, evolve it into a narrative with a particular point of view. The features of the group have been reported elsewhere [19]. While waiting for members to respond to their questions asked online over the two-hour meeting period, the participants were encouraged to doodle on their own, as they would have done in person as part of a group in the years before COVID-19—although there was no requirement to do so. Those doodles produced were shared at the end of the online meeting for interest, not evaluation. Participants were encouraged to see doodling as a way to pass the time while waiting to pose questions online to others and respond to questions provided to them.

To know their experiences with respect to the HeNReG at the end of each term, participants were asked to complete an online Google feedback form created by the facilitator following the model of the forms used by facilitators of other programs associated with the Health, Arts and Humanities Program in the Department of Psychiatry. The 2020/21 spring term was the first in which researchers were asked their opinion on doodling as an aspect of the group with the question, “What are your thoughts on the doodling aspect of the HeNReG experience?” To ensure that researchers provided an answer on the feedback form to the question about doodling, this question was tagged on the form as requiring an answer. It was the second time that researchers were asked how they believe COVID-19 has altered the HeNReG with the question, “Do you have other thoughts/comments on your experience as a participant in the HeNReG this term, especially as a result of COVID-19?” However, in order to ensure and answer was provided, spring 2021 was the first time their answer to the question was required, as a number of researchers had chosen not to answer the question on the fall feedback form.

The doodles of two participants who had both expressed persistent depression and anxiety with respect to their research were examined as part of a multi-year analysis that previously suggested doodling might be a measure of depression and anxiety [19]. Both participants joined the HeNReG during the 2017/18 academic year and continued with the group each year following—including during the 2020/21 year when meetings were no longer in person.

With respect to the doodles produced, it must be mentioned that some of the doodles may appear to be intentionally drawn. As such, it might be objected that the “doodles” produced by these researchers were actually planned efforts to create “art”. Experts in paleoart, for example, have argued that cave drawings cannot be considered doodles if there exist precise geometric patterns and an obvious symmetry to the drawings [33] or if there are shapes with a distinct, limited number of repeated motifs [34]. However, the reason for this pronouncement is the focus of these paleontologists on establishing the minimal semiotic capacity in their creators, not in examining criteria for the doodling of academic researchers. Although some of the doodles produced by the group participants may look like planned art where doodles produced in other situations do not, normally when people engage in doodling in other contexts they are not encouraged or given an extended period to doodle [35] as they are in the HeNReG. It was clear in the explanations that researchers provided of their work that their doodles developed throughout the session. Planning, evaluating, and forethought were not part of these creations, as they would be in deliberately making art. Instead, the doodles were drawn aimlessly, focusing on motifs often chosen by a particular participant when deciding to doodle or else reflecting the doodler’s immediate surroundings.

## 3. Results

In comparing online doodling during the time of the COVID-19 pandemic with the previous years, it is relevant to examine what group members thought of the doodling aspect of the program, the amount they actually doodled, and how they believed COVID-19 had affected the function of the HeNReG. What they thought about doodling, and the effect they believed COVID-19 had had, are results taken from the returned feedback forms. The amount each member actually doodled became available from examining the private Facebook group posted images for the 2020/21 academic year in comparison with previous years.

### 3.1. Feedback on Doodling and COVID-19

There were two participants who did not return the spring 2020/21 feedback form. One was a researcher who participated in the fall term only. As the doodling question was added during the second term, his view on doodling was not requested, although when he did participate in the fall he was one of the researchers who doodled. The other participant who did not return the feedback form indicated she, “wasn’t able to participate enough to be able to answer the questions. It’s been a tumultuous year at work.” The full responses of the members to the spring 2020/21 feedback form with respect to doodling and COVID-19 can be found in Table 1.

What is particularly interesting about the feedback results presented in Table 1 is that there were only two members who, when asked, “What are your thoughts on doodling aspect of the HeNReG experience?” provided a reply that focused on the fact that they themselves did not doodle. Why this is noteworthy is that only five members doodled more than five times throughout the course of the twenty-eight-week academic year, and seven members never doodled at all this year when the HeNReG was conducted online only. Yet, rather than mention that they themselves did not doodle as their response, the majority of the participants either said they found doodling somehow useful to them or that they really loved or enjoyed doodling.

### 3.2. Pre-COVID-19 Doodling in Participants Reporting Depression and Anxiety

Over the first three years of their membership to the HeNReG, two researchers told the group that they were experiencing anxiety and depression related to their research and that this was affecting their work. The doodles produced in these academic years by the two researchers were ones that gave reason to suppose that doodling may be an effective measure of disaffect. The reasons for this assessment were previously discussed and the doodles presented [19]. These particular doodles and the descriptions provided by the researchers regarding the doodles will be presented here again to make the comparison between them and the COVID-19 year to follow evident.

**Figure 1:**
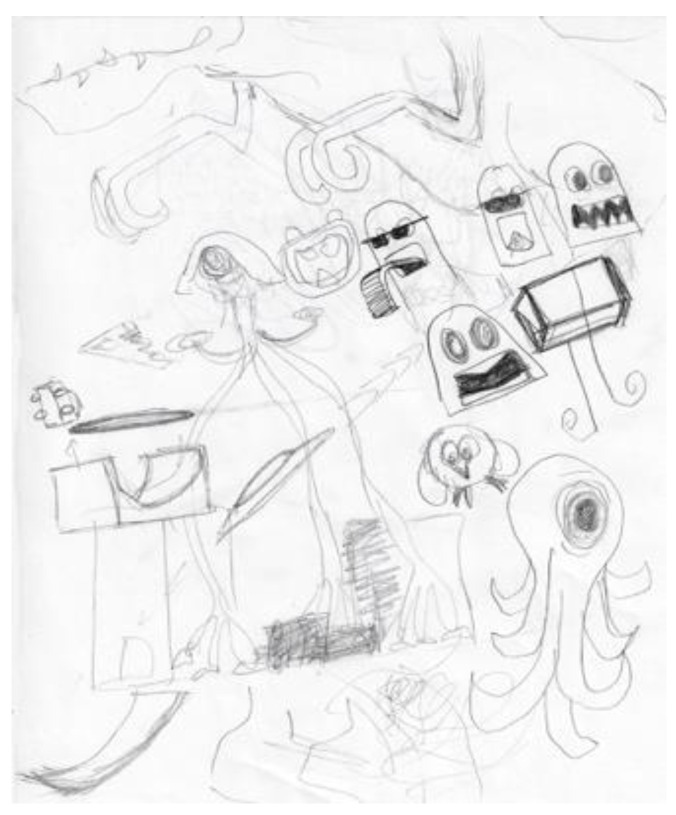
Doodle when participant was initially depressed and anxious about his research.

**Figure 2:**
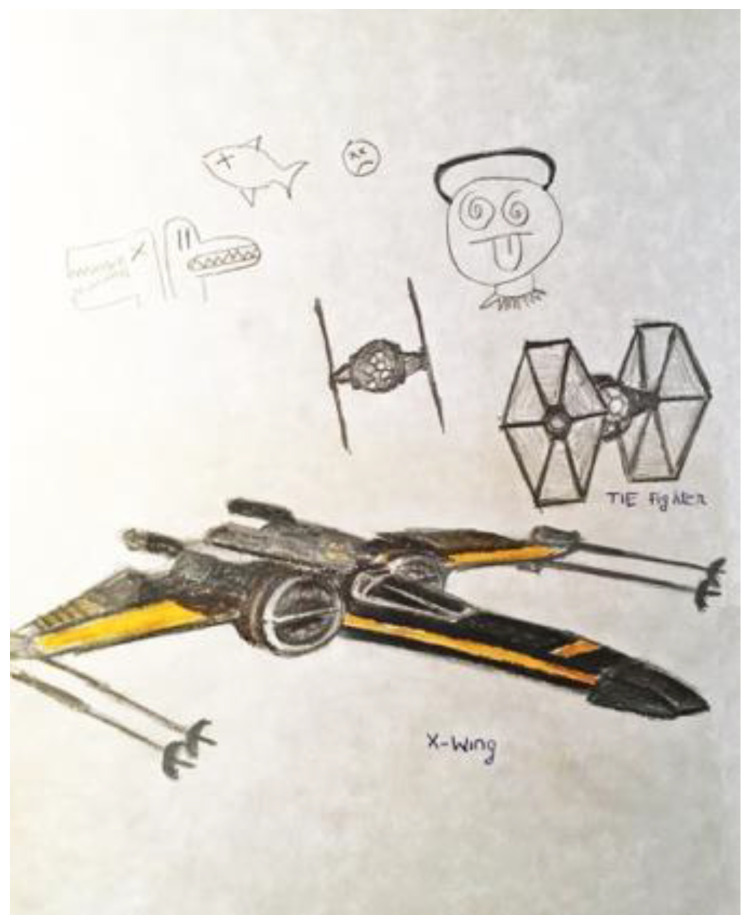
Doodle after participant switched disciplines for his research.

**Figure 3:**
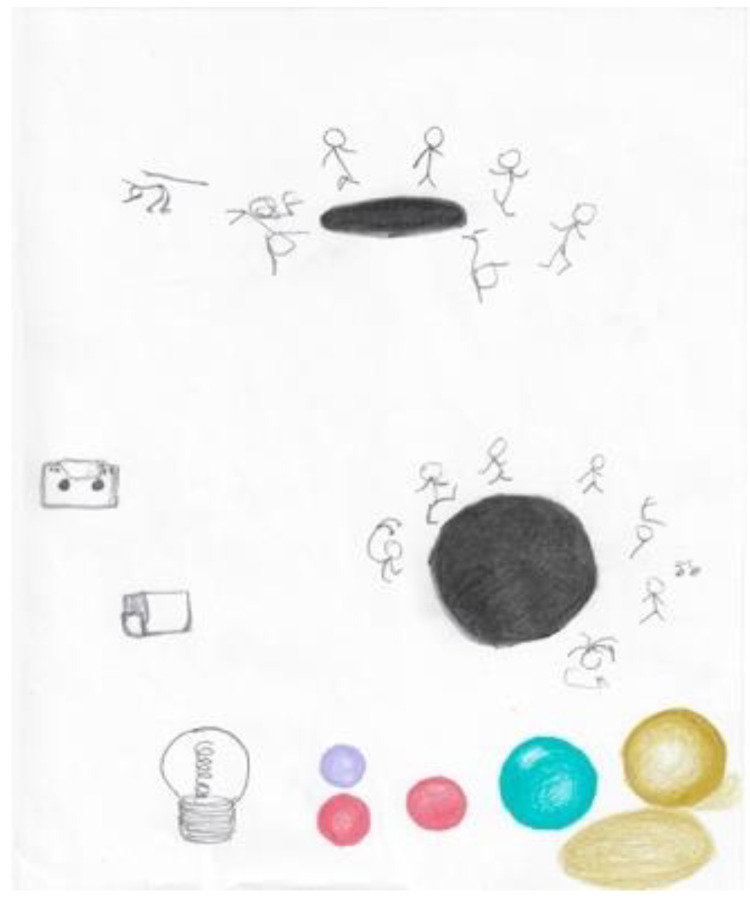
Doodles by participant after personal tragedy increased depression and anxiety.

**Figure 4:**
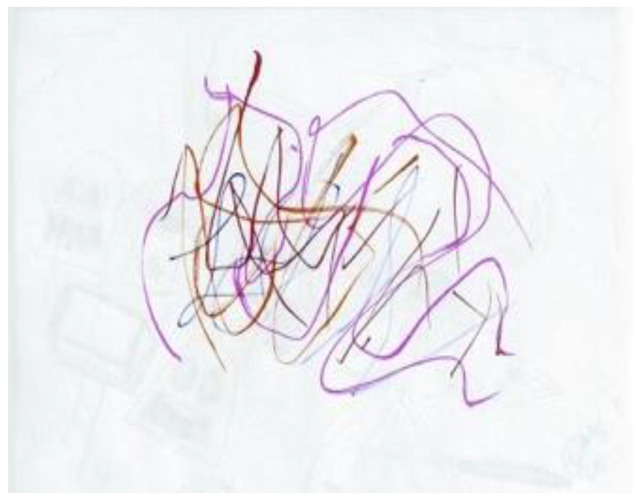
Doodle described by participant as “not feeling it” in using colors.

**Figure 5:**
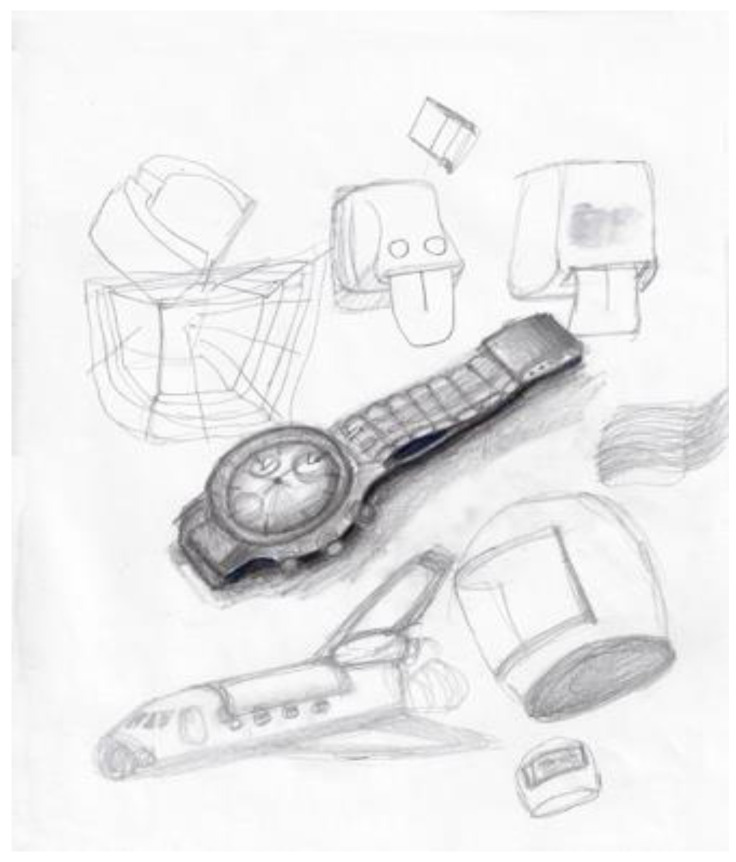
Doodle by participant focused on space and time.

**Figure 6:**
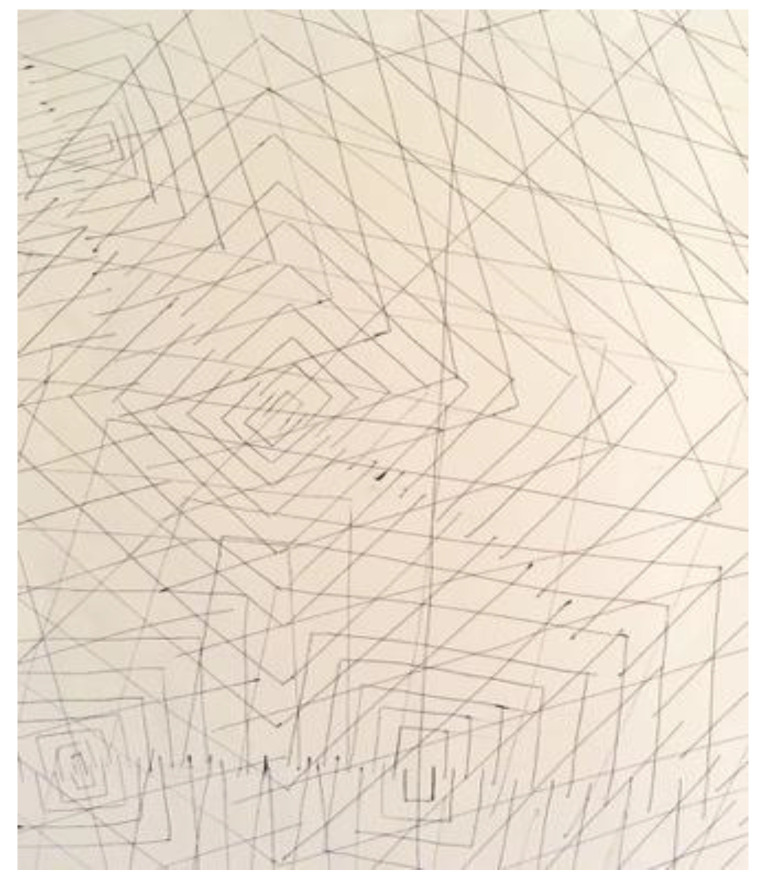
Doodle by participant of first abstract figure.

**Figure 7:**
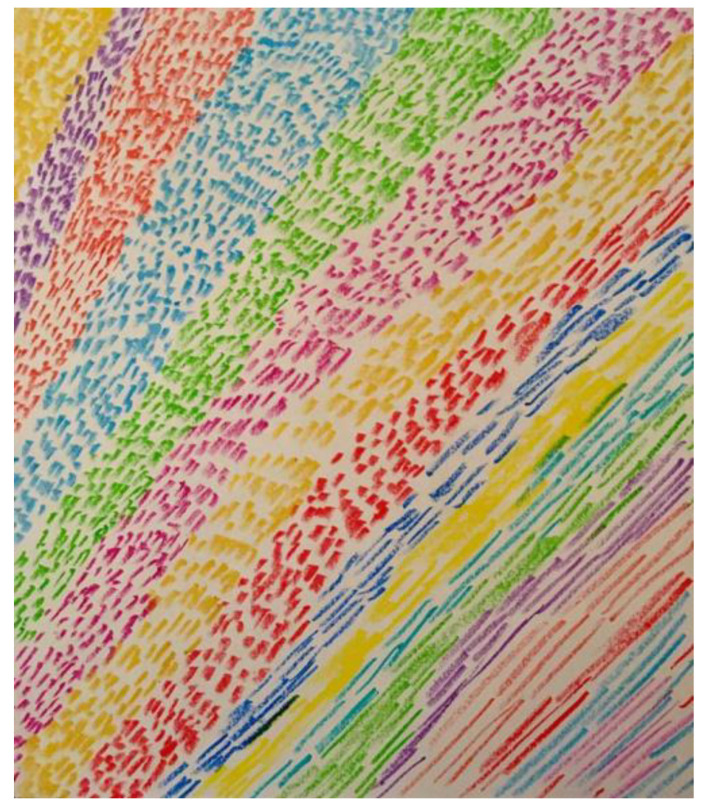
Doodle when participant focused on effect of color.

**Figure 8:**
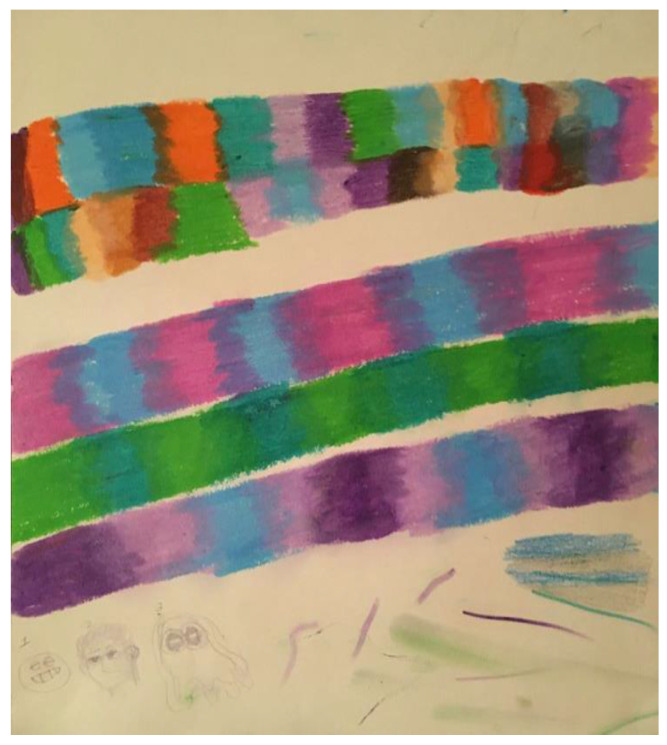
Doodle when participant wanted to have fun with color.

When initially depressed and anxious about his research, the first researcher’s doodles were of small, unrelated objects (Figure 1). Once he switched his research discipline, he began to produce larger doodles (Figure 2). After the personal tragedy that increased his depression and anxiety, he doodled his acquaintances dancing around a black hole (Figure 3). It was the first time black represented a prominent color in his doodles, that he drew people in a group, or he had produced stick figures. That same day, he drew a page of colorful squiggles (Figure 4) of which he said he “wasn’t feeling it.” In the weeks that followed, his doodles became focused on space and time (Figure 5). He then doodled his first abstract pattern (Figure 6) mentioning he was starting to feel better. After this, he started to focus on the effect of color in relation to his design (Figure 7). For the next doodle, he wanted to “have fun” blending colors (Figure 8) where now larger-page doodles no longer express identifiable content.

**Figure 9:**
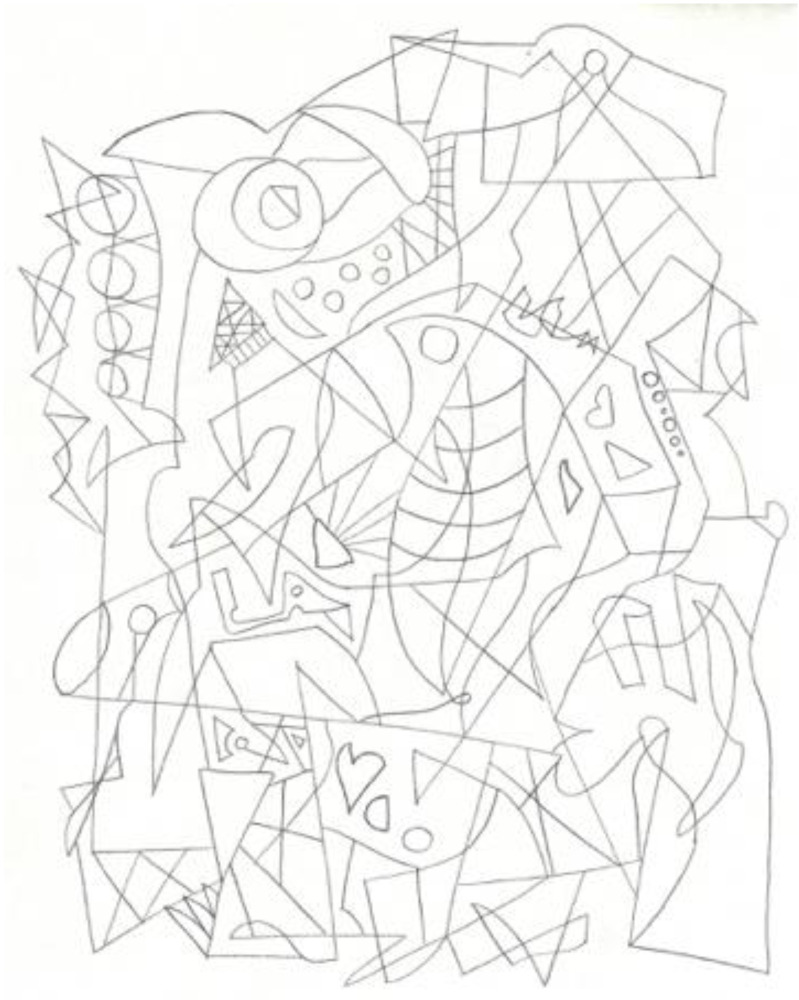
Doodle by participant of small parts arranged complexly.

**Figure 10:**
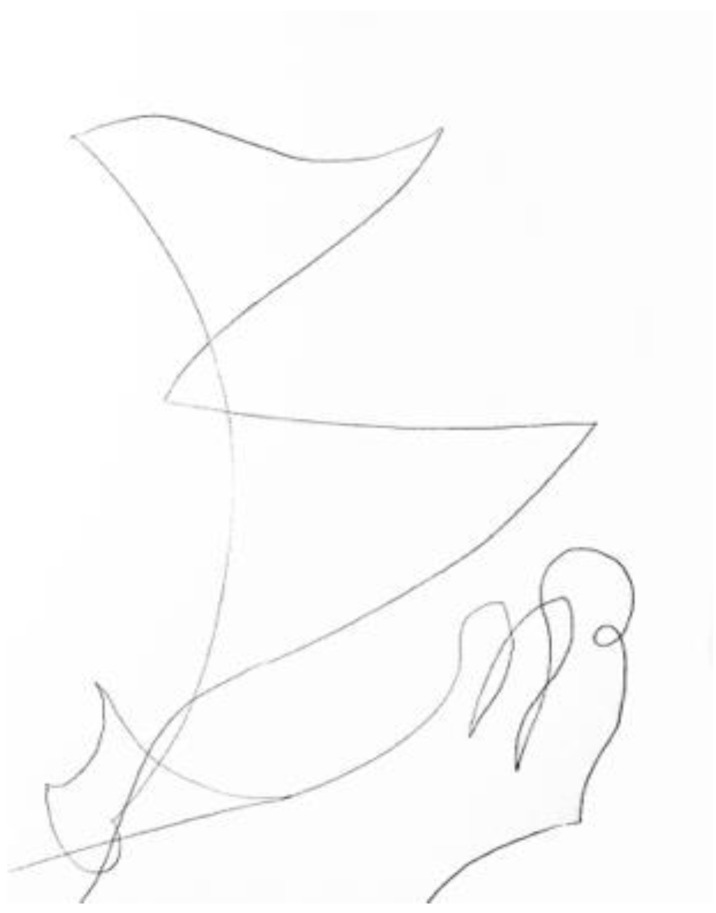
Doodle on the day the participant said he was losing energy.

**Figure 11:**
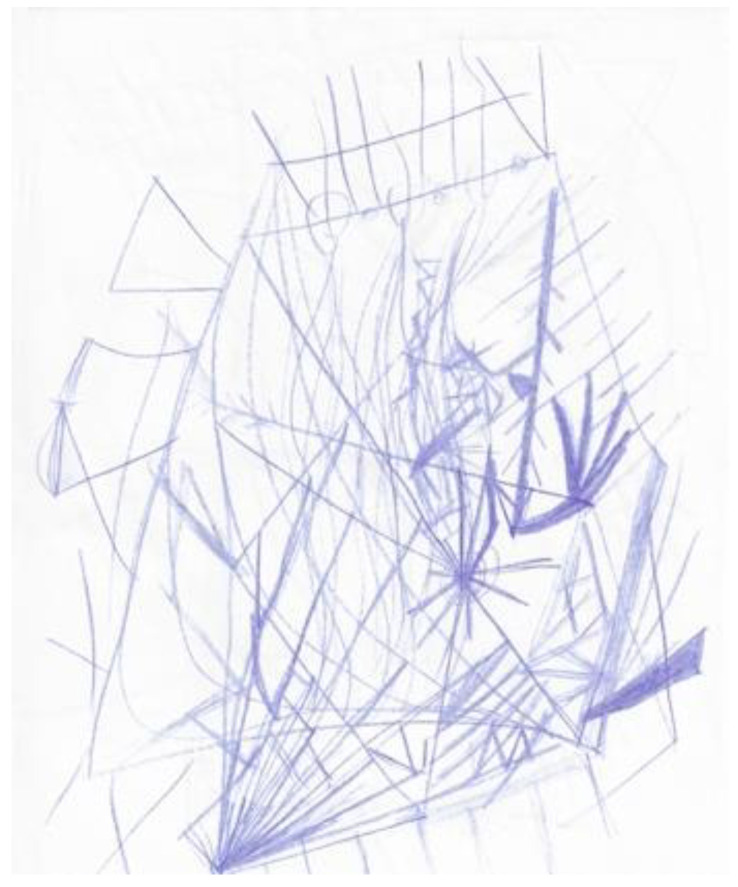
Doodle by participant expanding upwards from one point.

**Figure 12:**
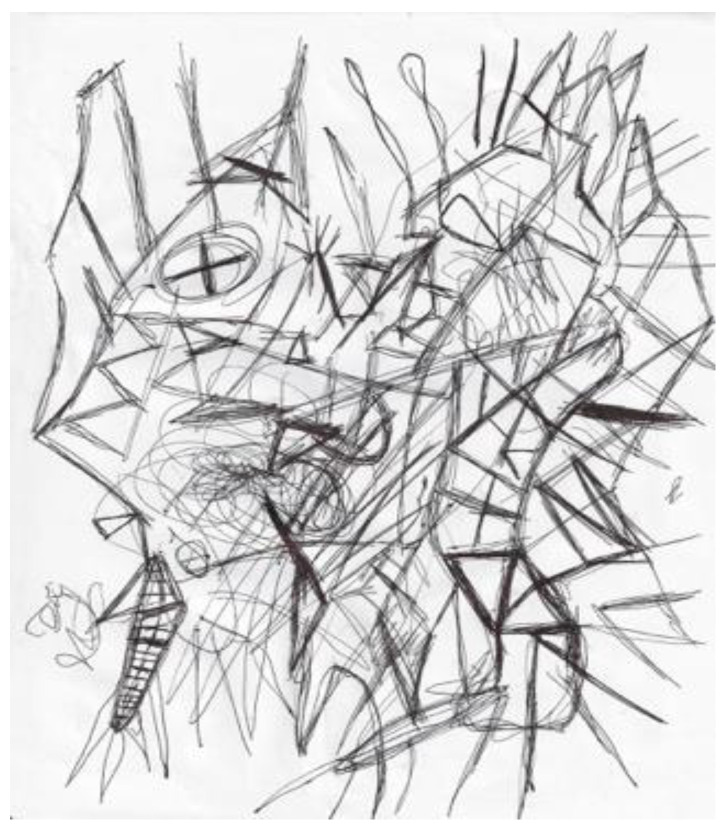
Doodle on the day the participant arrived obviously ill using heavy black lines.

**Figure 13:**
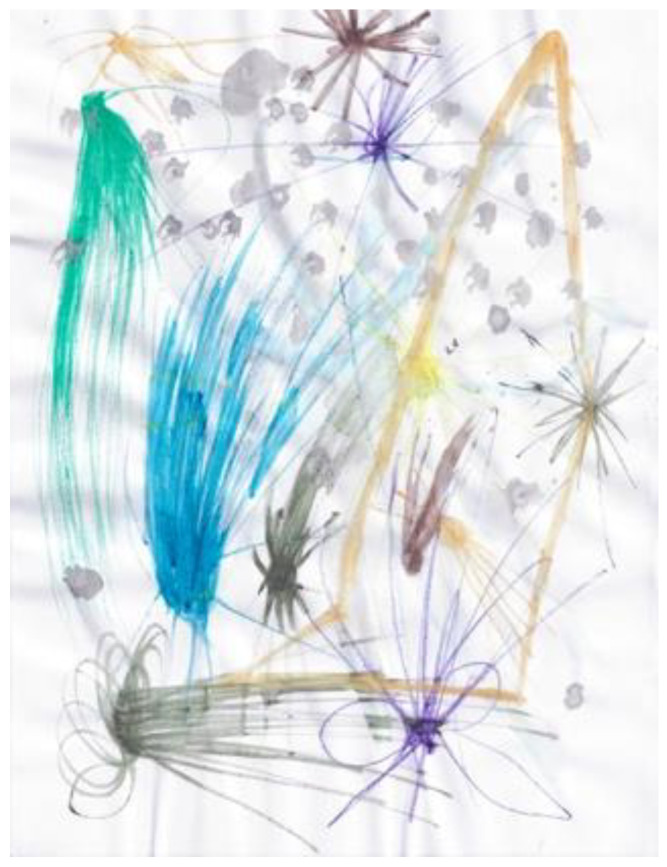
Doodle when the participant returned after an absence and he was “beginning to work things out”.

Another researcher was agitated and depressed regarding his lack of work progress when he joined the HeNReG in 2017. Similar to the previously discussed group member, the doodles he produced corresponded to his mental state. Initially, his doodles were of small complexly arranged parts (Figure 9). When he felt he was losing energy on another day, his doodling reflected this (Figure 10). With increasing reported depression, his doodles focused on expanding upwards from one point (Figure 11). When obviously ill, for the first and only time, his doodle used heavy black lines (Figure 12). After a three-month absence, his doodle included interconnecting ideas and colors created using a medium new to the researcher (Figure 13), which he described as representing that he was “beginning to work things out.”

There were only two members of the HeNReG who reported being affected by both depression and anxiety over more than a one-year period. Before COVID-19, the doodles of these two HeNReG participants attested to their internal mental state at the time each doodle was created. During these years, when they felt particularly depressed, this showed in the types of doodles both researchers created and how they chose and engaged with their materials. Intense depression brought with it doodles unique to that experience.

### 3.3. Doodling of Participants Reporting Depression and Anxiety during COVID-19

Without the casual conversation that characterized the team mindfulness of the in-person group meetings pre-COVID-19, many of the details of these two researchers’ depression and anxiety remained unshared during the 2020/21 online meetings. It was with respect to additional messages the facilitator received from these two researchers, outside the HeNReG meetings, that they provided information indicating that both continued to experience depression and anxiety with respect to their research, influenced by their isolation resulting from COVID-19.

The outcome for these two researchers during the pandemic year with respect to doodling was distinct from any previous year. One difference was that these researchers rarely doodled when they were left to doodle on their own. With in-person meetings, these two researchers enjoyed doodling at each HeNReG meeting. Now that they worked alone, they did not feel inclined to doodle.

Another dissimilarity from previous years was that the doodles they did produce were much less about the materials used for the doodle than they had been when, at the in-person meetings, various artists’ materials had been in easy reach of every researcher. Instead, the doodles they produced during COVID-19 were done exclusively with pencil using whatever paper was on hand for the researcher. In the case of the one participant, it meant all doodles were done in a small notebook. For the other, the one doodle produced for the entire year was done on a lined piece of paper intended for writing. The final difference was that none of the doodles produced for the year by these two researchers noted anything about their creators’ internal states. Instead, the doodles were records of what was in front of the researcher at the time. This meant drawing what was in the surroundings for one researcher. For the other, it entailed drawing over spills that had come through from the other side of the chosen piece of paper. In both cases, while doodling alone, the researchers’ mental states were obscured from what they were doodling in ways that they had not been in previous years.

**Figure 14:**
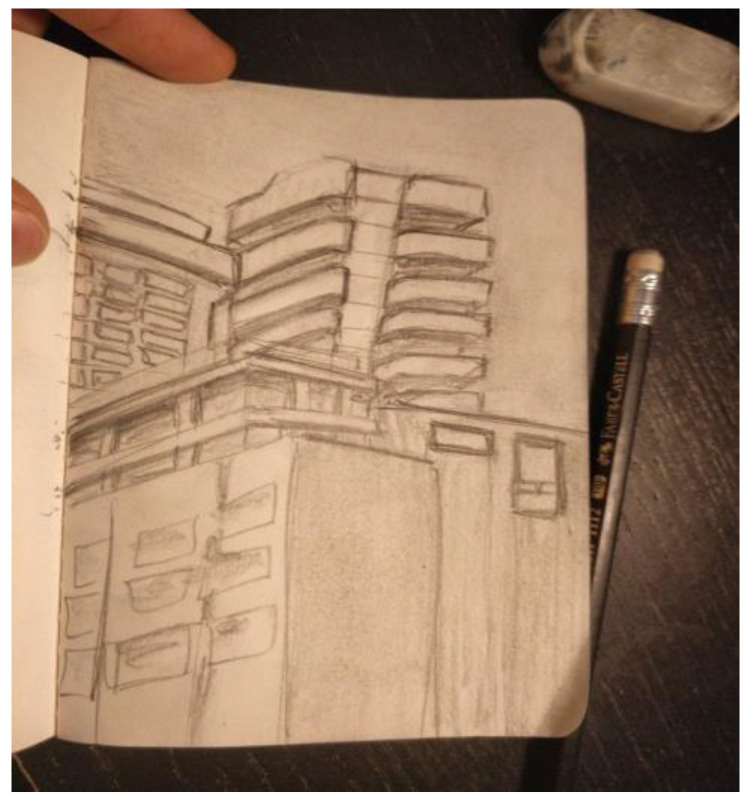
Doodle participant described as “the view outside my window”.

**Figure 15:**
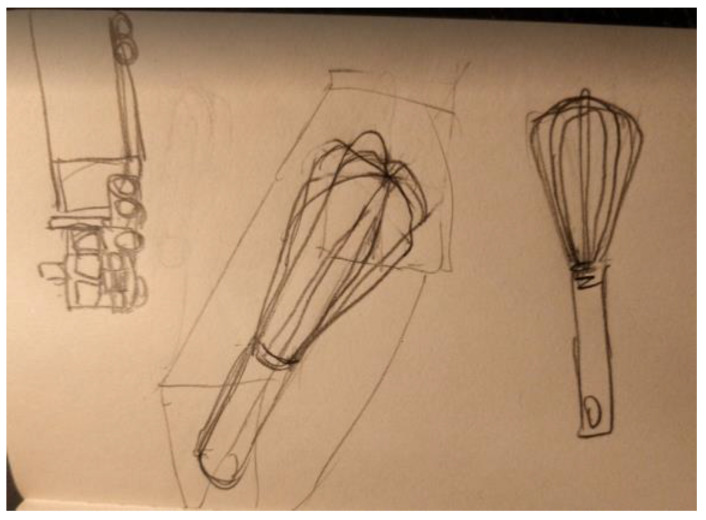
Doodle labeled by participant as “Truck and whisk”.

**Figure 16:**
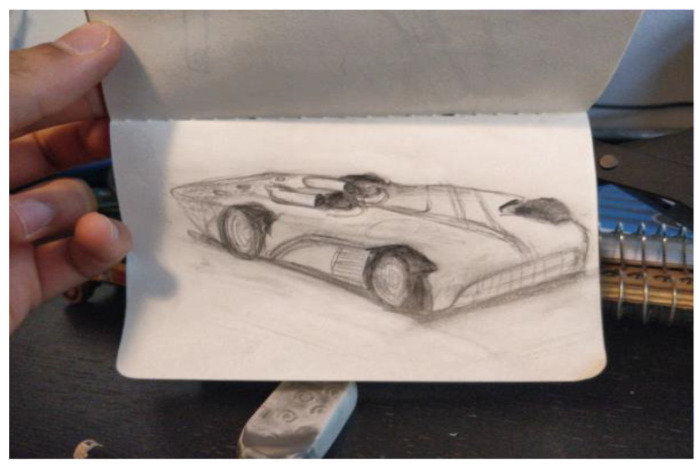
Doodle referred to as “It’s a Hot Wheels car I have” by participant.

**Figure 17:**
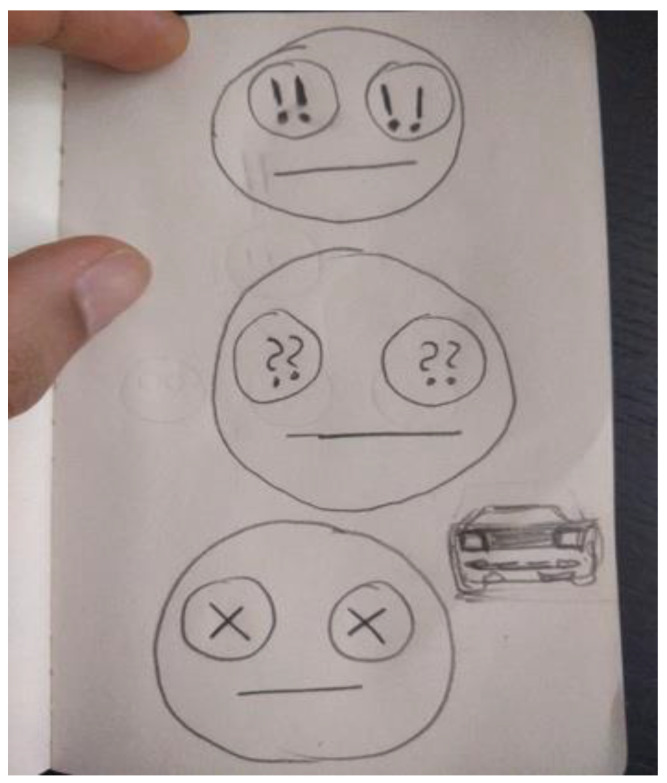
Doodle described by participant as “Three emotions of stress, and a Hot Wheels car”.

The first researcher made four doodles over the year. The doodles are presented here along with what the researcher wrote about them when the doodle was submitted to the facilitator for posting to the year’s private Facebook group. “It’s the view outside my window” (Figure 14). “Truck and whisk” (Figure 15). “It’s a Hot Wheels car I have” (Figure 16). “Three emotions of stress, and a Hot Wheels car” (Figure 17). Although the final doodle of the three emotions of stress may appear to be of something that recorded the inner psyche of the researcher, instead, the stylized drawings related to what the researcher had just been studying before the online HeNReG meeting. Although he contributed significant time to making each doodle, this participant did not include any noticeable degree of self-reflection regarding what he produced in his descriptions.

**Figure 18:**
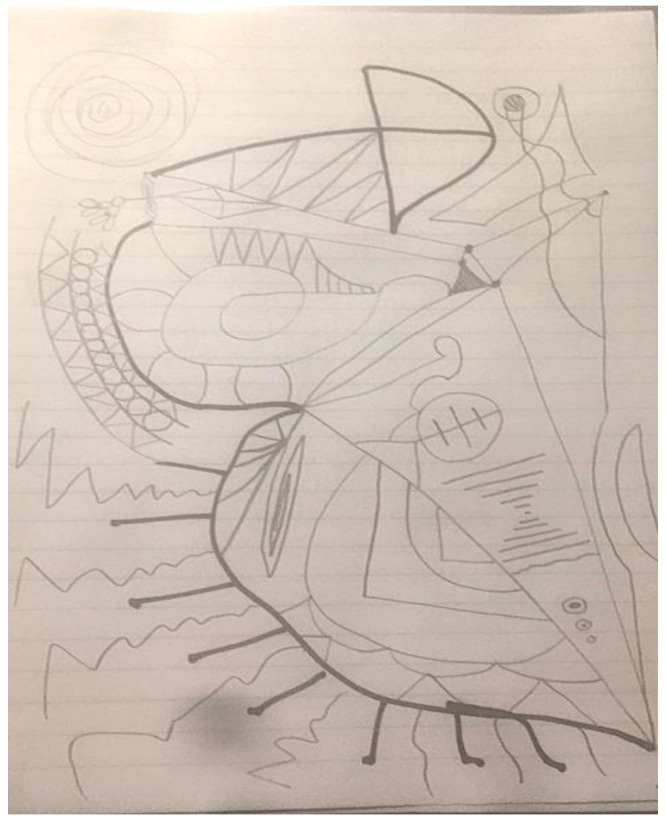
Doodle in which participant “just went with the flow”.

The second researcher completed only one doodle over the 2020/21 academic year, often stating to the facilitator that he was remiss in not producing a doodle on any particular week. On first viewing, the doodle that was produced seems in keeping with those produced in previous years. However, the description of the doodle indicates that, if there are similarities, they are coincidental. “Started off going with little spots where the marker ink from the previous page seeped on-to this page, and just went with the flow” (Figure 18). Thus, the doodle related to what was on the other side of the paper rather than the doodler’s internal states.

## 4. Discussion

In considering why most of the HeNReG members felt that they themselves did not have to doodle for the doodling to be a benefit to them, this may have been because the doodles of others who did participate in doodling were posted online each week at the end of the two-hour meeting for every member to see. It is possible that knowing doodling was permitted and seeing that it was always part of the weekly group were sufficient for group members to feel that the idea of doodling relaxed them. Yet, that so few members decided to doodle during the online meetings was, at the time, unanticipated by the facilitator, given that almost everyone doodled each meeting when the group meetings had taken place in person.

Regarding the two members who expressed that they continued to experience depression and anxiety with respect to their research, the most obvious reason why the doodles were unable to reflect the inner states of the researchers is that these participants did not do enough doodling over the academic to indicate any particular tendency. It is possible that if they had chosen to doodle more often during the year they would have produced doodles that revealed their mental states. On the other hand, what doodling they did do was not in keeping with the type of creations that might gauge any change in depression and anxiety for these two researchers.

One important reason why this behavior of group members with respect to doodling may have occurred is the underlying concern of all participants about the ongoing pandemic. A number of the researchers participating in the HeNReG were also frontline healthcare workers who were intimately affected by daily concerns regarding COVID-19. Yet, only two of the respondents were entirely focused on COVID-19 in answering the question, “Do you have other thoughts/comments on your experience as a participant in the HeNReG this term, especially as a result of COVID-19?” The others commented on the importance of the group for interaction during the pandemic. Most relevant to the discussion regarding doodling is that a concern with COVID-19 did not stop the participating researchers from thinking that doodling was a desired part of the group’s activities—yet the effects of COVID-19 may have been at least part of the reason why some people did not doodle themselves.

The paucity of doodling research makes the results comparing the previous years of the University of Toronto group with that of the pandemic year valuable. The progress from 2017–2021 of two doodlers in particular, with self-reporting changes to their depression and anxiety and considering doodling relaxing, was highlighted as examples of how impactful the limitations imposed by COVID-19 were in negating doodling’s ability to measure their depression and anxiety as it had for the three years previous. At least one of these two participants recognized this change in the relationship to doodling during the pandemic. The number 12 participant in Table 1 introspectively commented that doodling, “helps even more when we are doodling alongside others in the same room.” The other participant of the two who reported depression and anxiety, represented by number 2 in Table 1, reaffirmed the personal value of doodling regardless of the limitations: “I like it. It’s nice when I get to do it.”

### 4.1. Limitations

When the group had met in person, the facilitator had supplied various art materials in easy reach to encourage participants to doodle. Furthermore, those group members who knew how to use the different materials would demonstrate to others the techniques they had developed. In this way, an informal peer mentorship was provided by, and to, participants in how to use the art materials effectively. When doodling on their own, group members could make use of only those art materials that they themselves had on hand. If the participant did not normally doodle it would be unlikely that they would have the range of materials accessible to them that had been available in previous years at the in-person meetings. During those years, participants often commented that it was the intriguing art materials that encouraged them to want to doodle [19].

Another experience that in-person meetings previously provided to participants was the casual comments that would be made as people doodled. Someone doodling would say out loud, “I’m switching to using the creamy colors”, “I decided to make circles”, or “I don’t know why I decided to draw this.” None of this talk was deeply revealing or relevant to the discussion of the written work occurring concurrently at the time; however, what it did do was remind the other participants that they too could be doodling if they were not or, if they were already doodling, to keep up their efforts. This feature—indicating the team mindfulness of years before—was not available in 2020/21, as participants who doodled did so on their own at home.

Lastly, the part of the in-person meetings that participants often enjoyed the most was the end-of-meeting sharing of doodles that took place, when members would have the opportunity to talk about their doodles. At that time, other members often would ask questions of the doodler about their creation, providing additional opportunities for the doodler to mention what the doodle expressed in relation to their mental state at the time. It was not unusual for participants experiencing depression and/or anxiety to reflect on their mental states when talking about their doodle as a result of these in-person questions. This after-meeting sharing was an aspect to doodling that was lost when the sharing of the doodles took place only online. Although participants could have asked questions online of the doodlers in relation to what they had doodled, this never took place. In other words, the team mindfulness was no longer sufficient for group members to feel inclined to be self-reflective regarding their inner mental states.

The limitations mentioned above, either on their own or in combination, might explain why solitary doodling was unable to act as a measure for depression and anxiety in comparison to in-person, group doodling. As none of the participants provided sufficient detail to their comments about doodling and, in fact, seemed to lack insight into their doodling when having to engage in it on their own, conclusions that can be drawn need to take these limitations into consideration.

### 4.2. Measuring Burnout

Although doodling was not found to be a potential measure of burnout under the restrictions imposed by COVID-19 for this particular group, measures of burnout have been developed and studied by psychometric researchers with respect to COVID-19 in various settings. Examples of these include: the COVID-19 Burnout Scale (COVID-19-BS) —investigating the mediating effect of resilience on the relationship between COVID-19 stress and burnout [36]; the Copenhagen Burnout Inventory—measuring burnout, as well as depression, anxiety, distress, and stress [37]; and the Maslach Burnout Inventory-Human Services Survey (MBI-HSS)—assessing burnout directly while highlighting emotional exhaustion [38]. Nevertheless, none of these measures employed and examined during COVID-19 were tested on researchers per se, nor are they simple to administer, as doodling might be if it were found useful as a measure. There is little research on burnout in researchers in general. One study [39] published during COVID-19, but conducted before the pandemic, tested the usefulness of Achievement Goal Theory and found that burnout in researchers is related to normative performance avoidance goals, causing unfavorable goal setting processes. The intention of this study, however, was not to provide a measure of burnout in researchers. Instead, its purpose was to evaluate the usefulness of Achievement Goal Theory for describing research motivations, investigating which goals researchers pursue, and examine their associations with job burnout/engagement and professional learning. As such, the findings on burnout in researchers were evaluated from the perspective of achievement goals with respect to the rubric of this particular theory, and may not provide an entirely appropriate nor useful measure of burnout regarding researchers participating in research groups with the particular features of the Health Narratives Research Group.

## 5. Conclusions

In a health narratives research group organized to appeal to those indicating burnout from work-related depression and anxiety, a possible way to measure the participants’ change in depression and/or anxiety is intriguing [40], especially when comparing pre-COVID-19 years with the first full academic year of the pandemic. What has been identified is that researchers encouraged to doodle under these pandemic conditions, when asked for their feedback, are positive about the activity of doodling. Nevertheless, few of them doodled themselves. Therefore, it can be concluded that seeing the doodles of others posted to a private Facebook group was sufficient to make them believe they felt more relaxed in group participation online. However, when the doodles of those who had indicated that their depression and anxiety related to work were examined, unlike the years pre-COVID-19, the doodles were unable to measure the changes in depression and anxiety expressed by these researchers. Although the participants themselves may have seen no difference in comparison with previous years, doodling was seen to provide ineffective results for measuring depression and anxiety related to burnout in those researchers who doodle.

### Regarding Team Mindfulness

With respect to team mindfulness, the outcome of doodling in the setting of the HeNReG can be compared to the two dimensions of team mindfulness: receptive, open, and non-judgmental experiential processing; and aware attention to present perceptions [12]. During the years when the HeNReG was able to meet in person, both of these dimensions were evident in the function of the group. However, during COVID-19—when meetings were no longer conducted in person—the aware attention to present perceptions was lost to the group; although the receptive, open, and nonjudgmental experiential processing of the group’s interactions remained within the online meetings through the private Facebook group. This gives reason for why the participants believed that the doodling relaxed them and improved their interaction within the group while, at the same time, the doodles produced by those indicating depression and anxiety were unable to act as a measure of depression and anxiety related to burnout when the group met online only. The key component of aware attention to present perceptions that was lost in online meetings is likely the active listening [41] by the participants to each other’s in-person descriptions of their doodles.

During the COVID-19 2020/21 academic year restrictions, the private Facebook group was able to continue most of the pre-COVID-19 interactions when the group meetings were in-person. Yet, by being unable to replicate the aware attention to present perceptions—one of the two dimensions of team mindfulness—doodling behavior by those expressing depression and anxiety was unable to measure these inner mental states. If what is required is active listening to the in-person sharing of doodles, then it should not be expected that doodling can act as a measure of depression and anxiety in researchers when they are unable to interact in person. Regarding burnout, the online results of 2020/21 for the HeNReG show that, although doodling is unable to measure changes to depression and anxiety, doodling retains the ability to relax participants. The ability of doodling to relax participants during COVID-19 limitations retains its value with respect to burnout in researchers participating in groups expressing team mindfulness, such as the Health Narratives Research Group.

## Figures and Tables

**Table 1:** Responses on the spring 2021 feedback form by column of (1) all 20 HeNReG participants to (2) “What are your thoughts on the doodling aspect of the HeNReG experience?”; (3) the number of doodles each member shared with the group over the 2020/21 academic year; and (4) “Do you have other thoughts/comments on your experience as a participant in the HeNReG this term, especially as a result of COVID-19?”.

No.	Response to Doodling Question	Doodles Shared	Response to COVID-19 Question
1	On the days that I felt like I had an idea, the doodles were helpful. But sometimes I went to doodle and I froze because I wasn’t really sure what to draw. In these moments, it was more beneficial to think about the conversation happening and not focus on the doodles.	5	This group was a great way to network as a student new to research, especially with all of the restrictions placed by COVID-19. This school year has been very isolating and I have not been able to go to the campus as a student yet. It was nice to have a platform where I could meet new people who I would have otherwise never have connected with.
2	I think it helps, but it helps even more when we are doodling alongside others in the same room. However, I did not doodle much this time around and I will get to it for the next year.	1	I reminisce about the times before when we would be able to meet at Mt Sinai, especially where there’s some special touch to being with one another in person.
3	I stopped doodling years ago as I came to perceive it as a sign of not paying attention. Learned that it is a great way to gauge my mood and thoughts that I am bringing to the session.	16	My only experience with group was during COVID. Doing the group online supported my ability to attend as no travel and also to spend time reflecting during the sessions. Also, anxieties related to speaking in groups was not an area I was concerned with. Facebook as a platform was a bit challenging as refreshing my screen did not always bring updated postings. Also, wonder if a more dynamic platform would be considered or tips for navigating the platform.
4	I love doodling, it’s one of the best parts of the HeNReG. People aren’t encouraged to draw in everyday life and I think this is a great way to encourage it.	8	Although I miss in person meeting, online participation was done very well by [the facilitator]. The flexibility of meeting online is also a positive.
5	I believe that it gave me something to do during the two hour period of the meeting while waiting for people to participate online.	28	I was surprised that working entirely online affected the ability of people to participate in doodling to such a great extent. As well, I hadn’t anticipated that so few people would ask others questions.
6	Love it! Especially in person, as doodling has always helped me feel calmer and more present in group discussions.	7	Having participated in HeNReG both in person and online, I have to note that it has been much more difficult to engage online, likely due to accumulated tiredness from all work and social activities being in a virtual format since the beginning of the pandemic. But I did appreciate [the facilitator’s] accommodative format of not running HeNReG through a video call platform but rather having a set time for online Facebook discussion.
7	I like it a lot but I think it’s easier to do the doodling in-person than online	2	I like the online environment especially because I don’t need to travel to the room.
8	Great aspect—I would like to take advantage of this more in the future	0	Unfortunately, due to strains of COVID on my day to day to job, my capacity to actively participate this term was limited. I hope in future sessions, I can more actively participate
9	Gives me some time to think a while and sketch messy ideas in my mind	0	Hope COVID 19 ends soon
10	I love the doodling aspect, because it *helps me as a fidgety person*	0	I think the way of handling the entirely online group was done very well!
11	I get carried away with doodling sometimes	4	I like the flexibility and structure of the meetings online, which allows me to read and reflect on the responses anytime of the day.
12	I like it. It’s nice when I get to do it	4	no
13	It improves one’s thinking capability.	0	My experience was fantastic. I enjoyed the course of the programme. It was a period of learning for a young and burgeoning researcher like me.
14	I have not participated in this part	0	Wish I could engage more and more actively
15	Some of them look super amazing!	0	I have been remote before already so not too different. Would be good if the time can be after work hours though. Might be nice to have an interactive call section to share and answer questions?
16	I do not doodle	0	Not now
17	Relieving	2 (photos)	It got me active engaged during the period of strict lockdown
18	Good	1 (photo)	Connection with other co-workers is important
19	*Not a member second term when question asked*	5	I appreciated being able to participate virtually during the COVID-19 pandemic. I wish there could have been a little more interaction, perhaps using Teams or Zoom? (*fall 2020 response*)
20	*Did not return feedback form*	2 (photos)	*Did not return feedback form*

## Data Availability

Data available on request due to privacy restrictions. The data presented in this study are available on request from the corresponding author. The data are not publicly available due to privacy restrictions expected by participants when they agreed to join the Health Narratives Research Group.
